# Exploring adaptive expertise in residency: the (missed) opportunity of uncertainty

**DOI:** 10.1007/s10459-023-10241-y

**Published:** 2023-07-01

**Authors:** Maria Louise Gamborg, Maria Mylopoulos, Mimi Mehlsen, Charlotte Paltved, Peter Musaeus

**Affiliations:** 1 Centre for Educational Development (CED), Aarhus University, Trøjborgvej 82-82, Dk-8000 Aarhus C, Denmark; 2 MidtSim, Department of Clinical Medicine, Aarhus University, Hedeager 5, Dk-8200 Aarhus N, Denmark; 3 The Wilson Centre, Faculty of Medicine, University of Toronto, 200 Elizabeth Street, 1ES-565, Toronto, ON M5G 2C4 Canada; 4 Department of Psychology, Faculty of Business and Social Sciences, Aarhus University, Bartholins Allé 11, Dk-8000 Aarhus C, Denmark

**Keywords:** Adaptive expertise, Clinical decision-making, Emergency medicine, Ethnography, Geriatric medicine, Medical education, Qualitative research, Residency training

## Abstract

Preparing novice physicians for an unknown clinical future in healthcare is challenging. This is especially true for emergency departments (EDs) where the framework of adaptive expertise has gained traction. When medical graduates start residency in the ED, they must be supported in becoming adaptive experts. However, little is known about how residents can be supported in developing this adaptive expertise. This was a cognitive ethnographic study conducted at two Danish EDs. The data comprised 80 h of observations of 27 residents treating 32 geriatric patients. The purpose of this cognitive ethnographic study was to describe contextual factors that mediate how residents engage in adaptive practices when treating geriatric patients in the ED. Results showed that all residents fluidly engaged in both adaptive and routine practices, but they were challenged when engaging in adaptive practices in the face of uncertainty. Uncertainty was often observed when residents’ workflows were disrupted. Furthermore, results highlighted how residents construed professional identity and how this affected their ability to shift between routine and adaptive practices. Residents reported that they thought that they were expected to perform on par with their more experienced physician colleagues. This negatively impacted their ability to tolerate uncertainty and hindered the performance of adaptive practices. Thus, aligning clinical uncertainty with the premises of clinical work, is imperative for residents to develop adaptive expertise.

## Introduction

Physicians in acute settings are faced with unpredictable circumstances, diagnostic uncertainty and unknown aetiology of symptoms (Croskerry, 2014; Kovacs & Croskerry, 1999). Therefore residents face the challenge of developing a set of thinking and acting habits that will help them cope with uncertainty and form sound clinical decisions under unpredictable conditions (Elia et al., 2012).

Given the fast-paced and uncertain nature of the ED, novice decision-makers are more prone to cognitive errors than more experienced physicians (Croskerry, 2014). To counter these errors, researchers and practitioners have developed decision aids, such as algorithms, guidelines, and probability programmes (Costantino et al., 2014). However, these aids have their limits (Schriger et al., 2017). Hence there is a need not only to focus on the decision aids, but how residents can be scaffolded so that they develop competencies in clinical decision-making. Therefore, the objective of this study was to analyse residents’ adaptive practices in the emergency setting. Specifically, we focused on exploring how individual and contextual factors might impact residents’ engagement in adaptive practices when diagnosing patients in the ED.

### Medical expertise

Medical expertise can be described as proficient pattern recognition, which involves the use of hypothesis testing strategies. According to Elstein and co-workers’ classical view, expertise builds on the premise that good decision-making is a function of accumulation of knowledge and experience, and that difficulties in problem solving are due to insufficient knowledge and experience (Elstein & Schwarz, 2002; Elstein et al., 1990). Medical expertise is contextual and content specific (Elstein, 2009), and clinical judgement is vulnerable to biases and depends on heuristics (Norman, 2009; Norman & Eva, 2010). However, this classical understanding of expertise neither accounts for adaptability amongst novice physicians, nor concerns itself with the application of knowledge. In a rapidly developing healthcare system, adaptability and creative thinking have become imperative (Gube & Lajoie, 2020; Mylopoulos et al., 2016). The resident needs to prepare for an uncertain future, and as pattern-recognition is content and context-specific, it only prepares the learner for what is known at any given moment. It does not, however, prepare them for how to learn new skills or knowledge, which is important in ever-changing settings (Barnett, 2012). It is therefore important to understand how knowledge is organised and used (Eva, 2005), and how to promote adaptability amongst novices (Gube & Lajoie, 2020; Schwartz et al., 2005; Zimmerman, 2013).

Almost forty years ago, the concept of adaptive expertise was proposed by Hatano and colleagues (Hatano, 1982; Hatano & Inagaki, 1986). Based on Piaget’s epistemology of cognition as adaptation, assimilation and accommodation (Furth, 1981), Hatano and colleagues described a difference between a person’s procedural knowledge, which is knowledge of how to perform specific procedures, and conceptual knowledge, which is the act of giving meaning to procedural knowledge (Hatano & Inagaki, 1986, p. 2). This ‘giving meaning to’ can be used by the person to understand when and how to apply procedural skills by forming ‘mental models’ *“…with which people can run mental simulation, and thus make predictions/explanations about an unfamiliar object/situation…”* (Hatano & Inagaki, 1986, p. 2). According to Hatano (1982), as opposed to the routine expert, the adaptive expert can also adapt and invent new procedures to accommodate an unexpected situation. Importantly, adaptive expertise is the ability to both appropriately apply past procedural knowledge and develop new understandings based on conceptual knowledge when needed (Hatano, 1982; Hatano & Inagaki, 1986). There is a complementary balance between routine and adaptive practices (Bransford & Schwartz, 1999) where adaptive experts are assumed to be exceedingly proficient in both (Schwartz et al., 2005). Routine expertise relies solely on acquired procedural knowledge with no accommodation when the context demands a new solution. Currently, educational programmes are often structured in ways that promote routine practices of expertise. As a result, in order to fully understand how we can support the development of adaptive expertise during resident education, we were interested in gaining a better understanding of adaptive practices. Accordingly, in this study, we dichotomise routine and adaptive practices, and routine practices are therefore seen as disengagement of adaptive practices.

Hatano (1982) also suggests that curiosity and flexibility act as mediators of adaptive expertise. Conceptual knowledge can be gained through trial-and-error by simplifying the problem at hand. For example, when new residents associate an inverted position of a leg with the diagnosis of a fractured pelvis, they know the concept of a fractured hip and how this presents without an x-ray. They have, as such, distilled the diagnosis of a fractured hip to a specific symptom when paired with the knowledge that the patient had fallen. However, in complex cases, a flexible conceptual understanding is needed in order to construct ‘mental worlds’ and foresee complex interactions of patient conditions and related symptoms (Hatano, 1982). Here, conceptual knowledge is not enough. Adaptive expertise, according to Hatano, requires the continuous adaptation of new information to existing conceptual models. As such, while necessary, it is not solely the amount of conceptual knowledge that predicts adaptive expert behaviour but the degree of curiosity and flexibility the physician demonstrates as she critically appraises the applicability of her knowledge to a specific situation. Successfully diagnosing a fractured hip can be done without an expert understanding of orthopaedics. The success relies on the ability to effectively adapt to the unforeseen circumstances of the presentation of symptoms. As when the leg does not bend inward, as it typically would, but the resident, contrary to the guideline, orders an x-ray because they learn that the patient has fallen and knows that this could result in an unsuspected fracture.

In more recent studies (Mylopoulos et al., 2017; Van Der Schaaf et al., 2021), adaptive expertise has been described as a cognitive skill set that encompasses three approaches to knowing. First, epistemic distance is the person’s ability to distance his or her beliefs and knowledge from the problem at hand. Thus, adaptive experts are able to posit a separation between what constitutes their past conceptualisation of a problem and the emerging representation of this problem (Mylopoulos & Woods, 2009, p. 410). Second, self-regulation refers to the ability to continually observe one’s level of understanding of the problem space in order to regulate behaviour towards actions to improve understanding. Third, the ability to orient oneself to novel content and leverage opportunities for learning is a way for adaptive experts to improve their level of competence by improving existing skills and acquire new ones. As such, the key behaviour of adaptive experts is their ability to be aware of their knowledge and to accommodate to unknown situations by actively seeking out appropriate learning opportunities (Mylopoulos & Woods, 2009).

### Learning to perform as a physician

The construction of knowledge is dynamic and evolving rather than static and accumulative (Mylopoulos & Regehr, 2007, p. 1163), and it is an integral part of clinical decision-making. Therefore, it is important to understand how the social context mediates and moderate residents’ clinical judgement (Redelmeier et al., 2001). Newly graduated physicians face many challenges when starting residency as they begin taking primary responsibility for their patients. The experience of uncertainty is therefore a natural part of residency, but it is also a fundamental part of clinical practice throughout a medical doctor’s career. Uncertainty can arise when one’s knowledge of the observed, observable and unobservable world is insufficient (Djulbegovic et al., 2011) and has been linked to adaptive expertise (Cupido et al., 2022). In medical literature, uncertainty can refer to several things. Uncertainty can arise due to ambiguity in decision-making processes (Alam et al., 2017; Cristancho et al., 2013). It can also be related to self-doubt, arising due to insufficient or conflicting knowledge, or due to a lack of confidence in one’s ability to solve the problem at hand (Spafford et al., 2007). Djulbegovic et al., (2011) describes it as epistemic uncertainty referring to Fox’ definition, meaning that uncertainty arises from *“…incomplete or imperfect mastery of the existing knowledge… a consequence of limitation of the current knowledge… [or] a combination of the first two.”* (Djulbegovic et al., 2011, p. 321). In contrast, referencing Lonergan’s work, Engebretsen et al. (2016) argues that decision-making requires assessing information to gain new objective knowledge, and that this arises through novel events, which are inherently surprising and uncertain. They argue that uncertainty is thus important in decision-making, so long as it is accompanied by self-awareness:

Objectivity is self-appropriation. It is the result of a gaze turned inwards not outwards. Thus it does not exclude uncertainty but encompasses it. (Engebretsen et al., 2016, p. 598)

Turning one’s gaze inwards, requires one to doubt oneself. Thus, uncertainty as described as ambiguity cannot be isolated from self-doubt. This is further supported by studies, reporting correlations between low uncertainty (ambiguity) in diagnostic tasks and low anxiety, indicating an important psychological aspect of uncertainty, reaching beyond epistemic uncertainty. This is also underlined by Fox’ work on how students evolve in their ability to manage uncertainty, describing an initial anxious focus on individual limitations (Fox (1957) in Lingard et al., 2003).

Uncertainty has been linked to the development of professional identity. In their study on healthcare professionals’ communication during patient treatment and management, Lingard et al., (2003) depict how presenting and discussing uncertainties was an important socialising feature during case presentations for the development of professional identity. Other studies have further shown how such verbalisations help manage uncertainty, impacting residents’ chances of developing adaptive expertise (Apramian et al., 2015; Cristancho et al., 2013).

Despite the importance of managing clinical uncertainty, the continued ambition of standardising clinical work has led clinicians to negate the inevitability of uncertainty (Engebretsen et al., 2016). Uncertainty has come to be interpreted as an indication of incompetence (Ott et al., 2018), which has harmful implications for clinical training (Lingard et al., 2003). This is so, even if uncertainty is described as an integral part of clinical work, mediating the development of expertise (Cristancho et al., 2013). Thus, aligning professional identity and tolerance of uncertainty is important for adaptive expert development (Mylopoulos & Regehr, 2007). Mylopoulos and colleagues argue that, *“[a]daptive experts view themselves as ‘accomplished novices’ rather than ‘answer-filled experts’…”* (Mylopoulos & Woods, 2009, p. 410). This definition emphasises both the expert’s tolerance of uncertainty as well as a willingness to accept and expect knowledge gaps. The adaptive expert sees this as an opportunity to seek out learning opportunities to close these gaps. Thus, the development of adaptive expertise requires what has been termed ‘clinical courage’ (Fowler et al., 2021). However, being courageous is dependent on social structures allowing space for uncertainty and, therefore, the development of adaptive expertise is embedded in social structures. Although some scholarly attention has been devoted to the study of the development of adaptive expertise in educational settings, much less attention has been paid to how adaptive practices are influenced by clinical environments. (Kua et al., 2021). Thus, a need exists for investigating what mediates and moderate residents’ opportunity to perform adaptive practices.

## Methodology

### Design

This was a cognitive ethnographic study (Ball & Ormerod, 2000) of two EDs: one at a university hospital and one in a regional hospital. We followed Post Graduate Year 1 (PGY- 1) ED residents when they encountered geriatric patients, from the time of notification of a patient until a diagnosis was reached, and a treatment plan had been drawn up.

### Cognitive ethnography

The field of distributed cognition argues that cognition is inevitably imbedded in a social, cultural and physical world (Hutchins, 1995). With its origin in cultural-historical activity theory (Lave, 1988; Leont’ev, 1978), distributed cognition argues for the interchangeable, dynamic interaction between the individual’s cognition and the context (Hutchins, 2010), which is an uniquely human competency (Tomasello et al., 2005). By taking its point of departure in an ecological psychological approach, distributed cognition is a way of investigating how cognition is embedded in social practices and how affordances of the physical surroundings, such as social and technological means, shape cognition (Gibson, 1986; Hutchins, 1995). The method of cognitive ethnography aims to analyse distributed cognition and is a subfield of ethnography. The aim of this methodology is to use traditional ethnographic methods to explore participants’ interaction with their physical surroundings during cognitive tasks. Here, the aim is to both describe the meaning of social, cultural, and physical resources and to analyse processes of how these resources are employed during cognitive activities. As such, cognitive ethnographers organise observations based on an operationalisation of cognitive phenomena into specific episodes of activities. From these observations of episodes, the analysis aims to identify and explore which material and conceptual resources are present and how they are used in the construction of this specific cognitive activity (Williams, 2006).

Cognitive ethnography can be used to explore adaptive practices within broader discourses embedded in the clinical setting (Ball & Ormerod, 2000). The cognitive ethnographic approach is often utilised in clinical settings due to its more focused scope and shorter duration. This requires the researcher to specify observable behaviours that demonstrate the cognitive phenomenon studied.

As described above, the cognitive phenomena of interest in this study were adaptive practices. We therefore operationalised behaviours in the following subcategories: epistemic distance, self-regulation and orientation towards novel content. These operationalisations are described in Table 1. We analysed occurrences of activities, providing a narrative for how development of adaptive expertise was hindered or facilitated in the culture around residents in Danish EDs. To do this, we integrated observations with information from descriptions of cognitive artifacts in terms of objects within the physical milieu, such as patient journals, equipment, or technological aids. This allowed us to analyse how the culture of the ED facilitated clinical decision-making. Additionally, we continually performed clarifying inquiries, typically lasting five to twenty minutes, with the participants. This served to clarify thought processes and counteract possible misinterpretations of their actions. These inquiries were either performed at the end of the observation or during the observation at time points where it would not disturb the participants’ diagnostic process and interaction with patients, relatives, and colleagues.

**Table 1:** Operationalisation of the adaptive expert framework into adaptive practices

Cognitive phenomena	Theoretical definition	Examples of observable behavioural markers
Epistemic distance	The gap in knowledge representation between their present and past understanding of the problem	Looking up information, acts of surprise or simply stating 'I do not know'
		It could also be supervision from a supervising physician, questions from other staff or patients which illuminate gaps in the residents’ knowledge
Self-regulation	Residents’ display of self-regulation; monitoring current levels of knowledge and understanding, determining when this level of knowledge is not adequate for the problem they are trying to solve, and taking steps to address this gap	This could be ordering more tests or seeking help
		It could also be physical aids, organisational structures or other individuals, which makes regulation of residents’ own knowledge possible
Orientation to novel content	Orientation to novel content during problem solving: For adaptive experts, novel content presents an opportunity for learning in practice, a chance to fit their competence to the task rather than the other way around	Seeking learning opportunities, asking to follow other residents/physicians or following up on difficult patients to learn from their progress
		This could also be help-seeking opportunities such as other staff helping to see learning opportunities

### Participants

The primary investigator (MLG) observed 27 PGY-1 resident participants who treated, in all, 32 geriatric patients. Of these residents, nineteen were female, and they had on average 1,5 months of experience. Residents were recruited through a chief physician at the ED based on their availability on the day of observation. Transitioning into clinical practice and taking on the role of authority is challenging. Therefore, we chose to observe PGY-1 residents as we wanted to explore the starting point of adaptive practices to better understand how to structure postgraduate training which caters to the development of adaptive expertise. As described earlier, many medical education programmes promote the development of routine practices, and less is known about adaptive practices. Therefore, it was relevant to explore these practices amongst novices.

On the day of observation, the chief physician or the supervising physician would direct a geriatric patient to the recruited resident. Inclusion criteria for geriatric patients were that they be above the age of 60. During the observations, participants were observed to interact with eight fellow PGY-1 residents, 30 supervising physicians in introductory or specialist training, 33 nurses, 13 other healthcare professionals (emergency medical technicians, social workers, etc.) and 17 relatives or accompanying healthcare professionals. Data were collected from August 2019 until December 2020 with observation periods between 7 AM and 10 PM. The project was approved by directors at both departments. At one department, a chief specialist physician in charge of the PGY-1 residency programme also acted as a key informant.

### The Danish context: geriatric patients in emergency medicine

PGY-1 residents are important members of the day-to-day healthcare teams employed in Danish EDs. Alongside residents, the team of EM professionals consists of specialist physicians, medical students, nurses, nursing students, community care workers, medical laboratory technicians, physiotherapists, pharmacists, and secretaries. The team also includes residents and physicians from assisting departments, such as orthopaedic, lung, geriatric, and anaesthesia departments.

Geriatric patients are a growing population who make up a large percentage of patients in Danish EDs, and they are often characterised by the complexity of their health conditions (Franklin et al., 2011). These elderly patients are at higher risk of adverse outcomes (Galvin et al., 2017) than younger adult patients (Boltz et al., 2013; De Decker et al., 2014; Hwang & Morrison, 2007; Schumacher, 2005), often due to age-related frailty (Alexa, 2017; Laging et al., 2014; Melady, 2018). In addition, implicit ageism biases may impact decisions regarding geriatric patients (Cook et al., 2017). For this reason, investigating residents’ clinical decision-making in relation to geriatric patients in the ED was thought to be an ideal means of exploring the performance of adaptive practices. It is well researched that physicians make cognitive errors in EM in general (Croskerry, 2014) and with geriatric patients in particular (Galvin et al., 2017; Schumacher, 2005), making geriatric emergency care an appropriate context for the present study. Furthermore, recent studies has highlighted that intra- and inter-personal aspects of geriatric care is challenging to adaptive experts (Kua et al., 2022). It is thus relevant to investigate contextual circumstances for novices when engaging with this patient group. We operationalised geriatric patients in terms of age and accessibility. This choice was informed by the literature, which often defines geriatric patients as above the age of 65 years. However, in our study, we also included one patient who was 60 years old, as he presented as a geriatric case in terms of multimorbidity and frailty.

### Data

The primary investigator (MLG), who is a psychologist with two years of clinical training, had prior experience with various qualitative methods. She was supervised by PM, who had thorough experience with observational studies, both as a researcher and a supervisor.

MLG conducted 80 h of observations, taking the role of passive observer participant (Spradley, 1980), following the residents but not taking part in the diagnostic processes or patient encounters. She wrote field notes consisting of actions related to the concept of adaptive expertise. The field notes were guided by pre-defined observation cues (see Table 1: Operationalisation of the adaptive expert framework), notes from the clarifying inquiries and memos of analytical thoughts. Memos included reflections on the theoretical framework, common features observed among participants, changes to the operationalisation of the adaptive expert framework or similar notes relevant to the analysis. These notes were written out, just after data had been collected, as narrative descriptions of the entire patient encounter. These narrative descriptions consisted of: arrival notification, preparation, history uptake with the patient or relatives, consultation with specialist physician, ordering of tests, analysis, diagnosis, and treatment plan in consultation with a specialist physician, and handover to a nurse. Some days of observations included more than one PGY-1 resident, and these field notes were divided into separate, rich narrative descriptions (henceforth referred to as ‘observations’) of the patient encounter for each participant.

### Analysis: operationalisation of the adaptive expert framework

The first analytic step was to operationalise the construct of adaptive expertise in order to identify relevant observable behaviours (Ball & Ormerod, 2000). Based on the definitions of adaptive expert cognition provided by Mylopoulos and Woods (2009, p. 410), the primary investigator operationalised specific behavioural markers at both a contextual and an individual level. This operationalisation was then reviewed by two of the co-authors, PM and MM, where consensus was reached. MM is an experienced researcher within operationalisation of cognitive processes as adaptive expertise. The operationalisations were continually refined together with one co-author (PM) during data collection. The final operationalisations are described in Table 1.

The second step of the analysis was performed continuously during data collection, by MLG, PM and MM, and summative results were discussed by all authors in the author group during data analysis. We analysed each observation for adaptive practices based on the operationalisations (Table 1). Here, we coded each observation within the three cognitive constructs (epistemic distance, self-regulation and orientation to novel content) by sectioning the text and condensing interpretations of meaning within taxonomic tables for each construct (see Appendix 1). These taxonomic tables described three levels of analysis: firstly, who or what the resident interacted with (“actor”). Secondly, what were the material, cognitive or social moderators of their behaviour (“moderator”). Thirdly, what were the outcomes of this moderation (“consequence”).

## Results

The taxonomies for each cognitive construct provided insights on how different contextual elements, like how patients and electronic devices, could impact adaptive practices. The tables were structured to illustrate how an actor (e.g., the supervisor) moderated behaviours, and what impact that had on adaptive practices. We termed this impact ‘consequences’. We took the point of departure in the actors, as they were the main moderator of adaptive practices. Examples of these organizations of behaviours are illustrated in Table 2 below. For the full tables of each cognitive construct, please consult Appendix 1.

**Table 2:** Examples of taxonomy of adaptive expertise

Actor	Moderator	Consequence	Example
*Epistemic distance as adaptive practice*			
Patients and relatives	Use relatives to gain information	Explore the problem space	The resident (Daniel) concludes this encounter by stating a preliminary treatment plan and asks about the wife’s checklist. The wife hands the resident her phone, where there’s a text message from her daughter. The resident reads the message and asks: *“his head tilts towards the right?”*
*Self-regulation as adaptive practice*			
Electronic patient journal	Referrals	Cueing monitoring current levels of knowledge	While explaining how he uses referrals, the resident (Daniel) explains to the researcher: *“There’s also specific referrals which calls for specific actions. For example, “OBS pneumonia” or “OBS infection” are often more general indications of an overall worsened habitual condition and then you prepare for anything. Then, you are OBS on everything.”*
*Orientation to novel content as adaptive practice*			
Electronic patient journal (EPJ)	Test results	Identifying learning opportunities and acquiring help from specialists	The resident (Christina) looks at the CT scan and checks with pictures of a typical fracture on the jaws that she looked up earlier. She is puzzled by the picture. She has called for an orthopaedic surgeon to come into the ED to assess the patient, and now comments *“I want to go with [the orthopaedic surgeon] when she comes, just to learn.”*

**Fig. 1:**
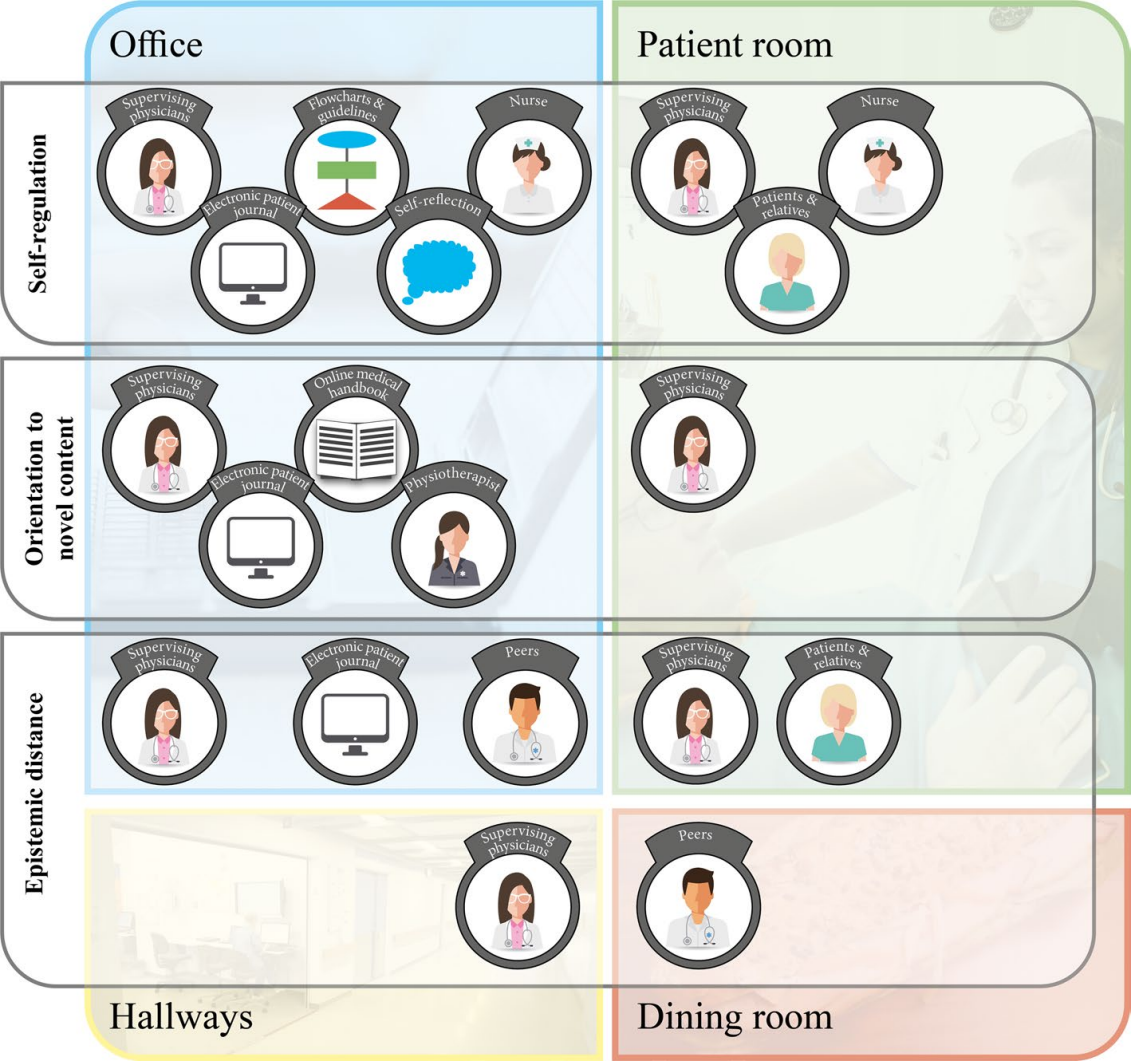
Adaptive practices distributed in the social and physical space. *The figure shows how residents’ adaptive practices were impacted by patients, relatives, artefacts (electronic patient journals, online medical handbooks, etc.), supervising physicians, peers, nurses and physiotherapists*

The taxonomic tables clearly showed that residents’ adaptive practices were both positively and negatively impacted by patients, relatives, artefacts (electronic patient journals, online medical handbooks, etc.), supervising physicians, peers, nurses, and physiotherapists (Fig. 1).

From the analysis that led to the taxonomic tables, two key moderating aspects of the social and physical space were identified; clinical uncertainty and decision-making disruptions. These aspects were pervasive across actors and often had a negative impact on adaptive practices.

### Clinical uncertainty

Uncertainty was often displayed by residents in the form of ordering more tests and a display of rigidity. Uncertainty was observed during all adaptive practices but was especially prevalent in self-regulation processes, as illustrated by the following excerpt:

Sophie (resident) is making decisions on how to get the best x-ray picture of the suspected fracture: *“I’m not sure if I should order both shoulder and upper arm, because I don’t know how specific they are… But I had one done on Saturday…”*. She looks up the x-ray from the other patient, and based on that, decides that an x-ray of only the shoulder would be adequate.

The excerpt illustrate how Sophie uses the electronic patient journal to self-regulate, when she encounters uncertainty.

However, uncertainty could also result in negative consequences, such as checking behaviours, as illustrated by the excerpt from Sarah’s case:

Sarah (resident) is treating her second patient of the day, whom has a possible fracture. She expresses needing more information for her to be able to decide and looks for a more experienced physician. When she cannot find one, she orders more X-rays to confirm her hypothesis before deciding on a treatment.

This excerpt illustrates that when a supervising physician is not available, it requires the resident to think on their own, which can infer uncertainty.

In terms of epistemic practices, several actors were in play. Supervising physicians could promote epistemic distancing by helping the resident gain a deeper understanding of the problem space by guiding them how to identify knowledge gaps. Here, residents would gain confidence in their exploration, which improved their sense of agency and adaptive practices. A few factors led to obstruction to the performance of epistemic practices. In some cases, the supervising physician seemed preoccupied with work that did not involve the resident. This led the resident to experience uncertainty because they missed the supervising physician’s support. In cases where the supervising physician continuously questioned the residents’ decisions, residents reacted by reinforcing checking and dependent behaviour. Here, checking behaviour is referring to using the supervisor, medical handbooks, nurses or peers to ‘check’ their thinking, decisions or opinions. In this study, most checking behaviours were asking their supervisor, if their decision or interpretation of symptoms was correct. The use of the electronic patient journal proved to be a key tool in prompting epistemic distance, both when the resident actively explored the problem space in focused read-throughs but also in regular preparatory discourses, helping residents detect knowledge gaps. Lastly, patients and relatives also impacted the residents’ ability to employ epistemic distance, both facilitating and hindering residents’ ability to maintain epistemic distance. Patients and relatives could both prompt identification of knowledge gaps (i.e., asking questions), but could also disrupt the resident’s thought processes.

With respect to the supervising physician, self-regulation behaviours were observed when the supervising physician supported resident agency, challenging them to reflect critically and model adaptive practices. Asking clarifying questions could decrease uncertainty and cultivate the residents’ behaviour of monitoring their own level of knowledge. The residents’ sense of agency seemed to diminish when the supervising physician adopted a more authoritative role, possibly because this hindered self-regulation and fostered dependent and checking behaviours.

Many helpful artefacts in the residents’ environment prompted and guided their adaptive practices; for example, electronic patient journals, online medical handbooks, laminated flowcharts and guidelines, and pocket booklets helped residents monitor their level of knowledge and decide which steps were relevant to take, in order to close knowledge gaps. The use of these tools was mainly prompted by patient complexity, uncertainty or when residents met unknown symptoms. Furthermore, referrals could cue self-regulating behaviour based on the expectation of knowledge gaps.

In general, behaviours demonstrating orientation towards novel content were sparsely observed amongst residents and were often related to uncertainty. These behaviours were primarily observed as a result of conferring with the supervising physician or other healthcare staff. The electronic patient journal acted as a tool to ensure future orientation towards novel content as residents would save patients and follow their outcomes; or, because of inconclusive medical tests, would acquire help from specialists and observe their decision-making in order to learn from their specialised knowledge. Another prompt could be when residents perceived that insufficient help was available from either online sources or supervising physicians, urging residents to actively seek out and identify learning opportunities.

During analysis of participant behaviours, a pattern of how residents oriented themselves towards a professional role was revealed as it impacted their adaptive practices. A lack of display of emotions and self-perception could be an indication of the high degree of perceived professionalism that was enacted in the culture of the ED. When questioned about this issue, residents referred to the importance of acting as a professional. However, supressing emotions had implications for how they tolerated uncertainty, as residents associated displaying emotions with unprofessional behaviour. However, registering and acting on one’s emotional response is an important function for tolerating uncertainty. This is both supported by literature, describing that adaptive expertise requires one to lean into uncertainty (Wineburg, 1998), which requires being comfortable with uncertainty and using negative emotions to guide and regulate one’s behaviours (Eva & Regehr, 2007).

A resident’s identity of being the senior to a medical student or in general being put in a superior, supervising or decision-maker role made residents gain confidence. This role, in turn, came with high expectations for residents to act as an expert, and was a fragile one that could easily be threatened. For instance, confidence and ability to act as an adaptive expert waned when a medical student questioned Sarah’s (a resident) hypothesis. Sarah failed to diagnose a fracture of the hip, and only succeeded in detecting it by prompt of the medical student. Her surprise of being wrong made her uncertain. This, in turn, prompted her to subsequently employ checking behaviour. Thus, her inability to meet the medical student’s expectations that she would perform just as well as her more experienced colleagues and not show uncertainty led to disengagement of adaptive practices. Moreover, Sarah abandoned her role as a supervisor for the medical student, being entirely preoccupied with the process of finding the right diagnosis.

### Decision-making disruptions

Disruptions could be described as interactions that made residents stop their decision-making process. They were characterised by displays of overlooking contextual cues, checking their decisions with available colleagues, or enforcing a rigid structure. Disruptions impacted both epistemic and self-regulated practices. They could for example, lead residents to possibly overlook important cues, as demonstrated in the excerpt from Catherine’s case:

Catherine (resident) is gathering anamneses on a patient whom she has difficulty establishing a contact, as the patient seems confused and distant. She starts to question the daughter about her mothers’ medication and chronic obstructive pulmonary disease. The daughter replies and mentions the patients’ memory problems. The patient interrupts: *“I feel unkempt… I have for some time.”*

The resident tries to calm her, saying that she doesn’t see her as unkempt at all. The patient looks lost, and it seems to me that the patient is trying to communicate a general discomfort or underlying problem, which the resident doesn’t pick up on.

However, disruptions could also have positive impact on adaptive practices. The following excerpt illustrates how disruptions from a lack of alignment between the patient’s symptoms and her working hypothesis, helped Christina to self-regulate:

Cued by contradicting findings in the physical examination of the patient’s pain, Christina (resident) re-examines the patients’ swelling. She wants to clarify if he is experiencing any pain in his face, other than locally from where he has broken his jaw and looks under his tongue for any haemorrhages. Christina comments that *“I do not think you have any new fractures; I think it is the swelling”*. However, she is not satisfied with the adequacy of her own knowledge and calls a specialist to confer her findings.

As with uncertainty, residents could experience disruptions from several actors and this affected all adaptive practices in both positive and negative ways. In terms of epistemic distance, relatives’ or patients’ behaviours disrupted residents, which could prompt them to further explore the problem space or make them overlook relevant symptomatic cues.

In terms of self-regulation, disruptions prompted residents to monitor their knowledge levels and actively take steps to address knowledge gaps by ordering more tests, doing additional physical examinations or seek out help from other medical staff. Nurses played an important role in residents’ ability to monitor knowledge levels and closing knowledge gaps by engaging in knowledge sharing and consulting situations. However, other healthcare staff could also hinder the residents’ ability to self-regulate. This would typically happen when they disturbed the patient or interrupted the resident. When this happened, the resident enforced a more rigid structure of history taking to keep track of their progression.

### The interplay between disruptions and uncertainty

By doing a narrative analysis of interactions between the residents and actors, we frequently observed an interplay between disruptions and uncertainty. The following case vignette demonstrates one example of how disruptions could affect how residents handled information and how disruptions impacted residents’ ability to orient themselves towards novel content and learning opportunities.

**Case vignette: Example of relation between disruptions and uncertainty**

*Participant: PGY-1 resident “Julie” (less than one month of training)*

Julie was treating a 91-year-old female patient who had been admitted because of dyspnoea. While Julie was trying to obtain a history from the patient, she was continually disturbed by other healthcare professionals who were there to draw blood samples, trying (and failing) to place a catheter. This caused increasing pain and frustration in the patient. The level of chaos in the history uptake then carried over to the conferral with the supervising physician which became sporadic and unfocused as Julie had a hard time sorting out the relevant information and generating hypotheses. As such, her approach to the conferring and the lack of help in organising and prioritising the information from the supervising physician did not initiate a shared reflective practice but rather became more similar to a handover, resulting in Julie the supervising physician just telling Julie what to do. From then on, Julie articulated and displayed uncertainty by checking all her decisions with the supervising physician.

**Fig. 2:**
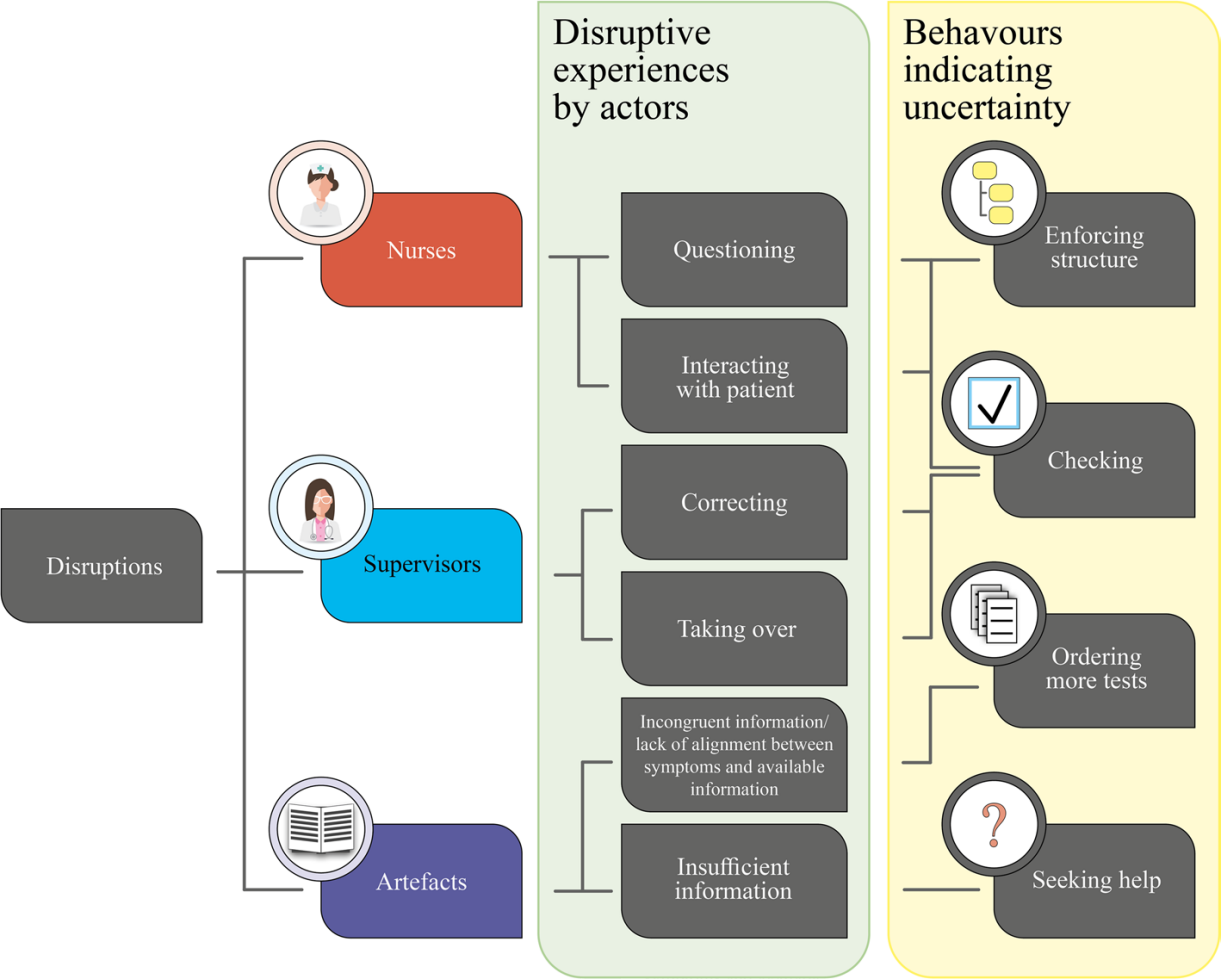
Interplay between disruptions and uncertainty. *The figure illustrates the interaction between observed disruptions from different actors and residents’ behaviours indicating uncertainty*

As exemplified by the above example, disruption of contextual learning could often trigger uncertainty. This was mainly observed when nurses or supervising physicians interrupted residents’ line of thought, or if test results or available information did not fit their working hypothesis. The interaction between observed disruptions from different actors and residents’ behaviours indicating uncertainty is illustrated in Fig. 2.

As demonstrated in Fig. 2, checking behaviour was often indicative of uncertainty and the ‘abandonment’ of adaptive practices. For example, continuous checking behaviour could be instigated or enforced by supervising physicians when they too strongly curated the patient meeting or completely took over the patient case. This often reduced resident autonomy, making them more dependent on the supervising physician and less inclined to make independent decisions.

## Discussion

The observed behavioural markers allowed us to observe the complexity of how residents performed adaptive practices throughout the geriatric patient encounter in EDs. It allowed us to understand the interaction between residents and their environment when residents made clinical decisions. The findings can be summarised in three main issues characterising workplace learning. Firstly, all residents engaged in adaptive practices, but they faltered when they faced uncertainty caused by interruptions. Secondly, how the resident perceived their role as an authority and accepted their own uncertainty, impacted their ability to handle uncertainty and perform adaptive practices. Thirdly, how the environment tolerated and supported the residents through this uncertainty was crucial to counteract inappropriate performance of routine practices.

### Adaptive practices amongst residents

In this study, all residents engaged in adaptive practices. In conformity with existing evidence, epistemic distance, self-regulation and orientation towards novel content are not separate, static processes (Mylopoulos & Regehr, 2011). Our findings convey an understanding of adaptive expertise as a dynamic and fluid capability. This means that the medical practitioner is not an adaptive expert in every clinical circumstance. Rather, the practitioner engages in adaptive practices by shifting focus when facing uncertainty. This observation further supports the argument that uncertainty does not indicate incompetence (Ott et al., 2018) and echoes findings from a recent meta review, that adaptive practices are engaged when physicians are faced with a change in their environment (Pelgrim et al., 2022). In this study, we added nuance to this picture, in that such changes could result in unproductive uncertainty. It is important to note that adaptive expertise relies on the accumulation of experience and knowledge, but that it is possible to engage in adaptive practices when being a novice. Physicians actively engaging in adaptive practices have the potential to develop adaptive expertise, over time. At the same time, routine practices serve a vital function in daily clinical work and are therefore a complementary dimension of adaptive expertise (Schwartz et al., 2005). A recent study explored routine practices amongst surgical residents, finding that standardization of practice was not feasible, but suggested that stabilization was a more feasible educational focus (Ott et al., 2021). This study builds this argument even further, suggesting that stabilization requires both routine and adaptive practices, as residents will then also be able to manage the inevitable novel problems they will encounter in their work, leading to overall stable performance.

### Professional role and uncertainty

The high demand for professionalism requires residents to assume the role of authority from their first day of residency. In this study, this perceived expectation was shown to lower their tolerance of uncertainty as uncertainty often did not align with how authoritative behaviour was modelled by more experienced physicians, or how they thought an authority was expected to act. If residents were able to counter uncertainty with curiosity, they were often able to act adaptively. This was also a good strategy when the context disturbed and disrupted their decision-making process It speaks to the importance of reconciling the authoritative expert role (Lingard et al., 2003) with the inevitable uncertainty of clinical practice (Engebretsen et al., 2016) when learning to become an adaptive expert (Apramian et al., 2015).

### How contextual support can mediate residents’ tolerance of uncertainty

In this study, residents who were more confident in their skills, while tolerant of uncertainty, did not rely on interactions with other healthcare professionals to guide and cue adaptive practices. On the other hand, residents who were less tolerant of uncertainty and less confident in their role as an ‘expert’ seemed to need external validation to find certainty, which then either came from checking behaviour (seeking validation amongst experienced staff), security measures (mindlessly asking for tests and information) or anchoring to their own existing beliefs. It is therefore imperative for residency training that the context supports the alignment between uncertainty and professional identity.

This study demonstrated the importance of supervisors performing uncertainty in the clinical settings, supporting earlier studies that also stress the importance of fostering a culture of acceptance of uncertainty to improve patient safety (Cristancho et al., 2013; Lingard et al., 2003; Ott et al., 2018). Residents were more adaptive when they were allowed to be uncertain, and when this uncertainty did not impact how the context viewed their ability to be an authority. As such, this study shines light on how supervising practices and resident’s understanding of what professionals ‘do’ had an impact on their ability to develop adaptive expertise (Rowland, 2017; Rowland et al., 2020). The study describes the negative consequences for learning when this expectation is present. The consequences were that residents were overpowered by uncertainty or forced to be overly sure of their diagnosis, which bore the risk of them making errors because they were not supported in acting as adaptive experts. This problem is especially unfortunate when uncertainty tolerance is linked with several desired healthcare-related outcomes amongst trainees (Strout et al., 2018), and uncertainty-related anxiety has a negative impact on the clinician’s own satisfaction with their decision-making competency (Libert et al., 2016). Here, studies argue that introducing a vocabulary for uncertainty in professional settings can mediate learning at all levels of experience (Lingard et al., 2003; Rowland, 2017). Uncertainty plays a role in developing adaptive practices and could be mediated through targeted verbalisation. LaDonna et al. (2017) described how learners are pushed to stage their performance in accordance with ‘textbook’ approaches when being watched. This hinders their ability to learn to act as adaptive experts when they are assessed in real clinical settings. Through an analysis of uncertainty rhetoric, Spafford and colleagues showed differences in novices’ and experts’ uncertainty, and how expert uncertainty made room for adaptations and innovations in their clinical work (Spafford et al., 2006, 2007). The present study underlines the importance of identifying and verbalising uncertainty in order for clinicians to act as adaptive experts.

The question of uncertainty is especially relevant when discussing how we train residents and, more importantly, how we assess them. A recent article by ten Cate et al. (2021) discusses the educational benefits of using entrustable professional activities as a tool of assessment, arguing that through this practice of workplace-based assessment, education and training will naturally act as a framework for ‘entrustment decision making’ (ten Cate et al., 2021). This speaks to the problem of acceptance of the inevitable risk involved in workplace-based training; namely that learning to adopt adaptive practices presupposes that the supervisor extends agency and trust towards the resident. However, this framework of entrustment decision making does not consider the importance of incorporating a curriculum for uncertainty.

### Limitations

While cognitive ethnography was a deliberate choice to critically and thoroughly investigate how cultures of residency training impacted learning adaptive expertise, it is important to highlight the specificity of our data. While the Danish clinical context bears some comparability to other international clinical contexts, some elements were also specific. For example, in these two particular EDs, all residents were required to confer all patients with a supervisor before moving on with the treatment plan. As such, supervision played a large role in residency training at these departments, and supervising physicians took pride in having this workflow implemented as a way of ‘taking care’ of their residents. However, such an arrangement is particular to these departments and is not implemented in all EDs in Denmark.

The choice of PGY-1 residents and the case of geriatric patients also carries some limitations. Several potential challenges come with treating elderly patients, such as communication and the complexity of their condition; and were chosen for these very reasons. In some instances, the patient was not able to cooperate. This made observations difficult like in the case of a patient with dementia who had expressive aphasia. This choice also excluded some good opportunities for observation of challenging situations of patients younger than 60 years old. However, complexity was argued to be important, and geriatric patients provided such complexity. Furthermore, PGY-1 residents never bore the full responsibility, and this could affect the observations and might explain why some residents were quick to seek help.

Lastly, some risks and limitations in regard to the cognitive ethnographic method were identified. Observer effects in ethnographic studies have been widely discussed, and it has recently been argued that they are not as prominent an issue as previously argued (Bressers et al., 2019; Monahan & Fisher, 2010; Varpio et al., 2017). However, given the specificity of the observations, interfering in the ecological setting of the participants’ distributed cognition may have an effect on the results. While MLG did not disclose to the participant the specific observed cognitive behaviours, she was obliged to informed participants of the aim of the observations. By disclosing this, she may have affected the ecology of their cognitive behaviour and have increased their reflexivity, which would be coded as adaptive practices. During the analysis, however, increased reflexivity or adaptive practices were observed neither in the beginning of the observations nor during events where the participant was especially engaged with MLG, which may indicate that this suggested effect is at a minimum. Furthermore, reliability was enhanced by including more than one investigator in the analytic process, which ensured that such effects were identified.

## Conclusion

One of the questions that research on adaptive expertise has grappled with is the role of uncertainty in the development of adaptive expertise. This study found that residents learned to engage in both routine and adaptive practices when faced with uncertainty. Adaptive practices were observed amongst all residents to various degrees and were seen as fluid rather than static; i.e., they occurred under some conditions and were absent in other situations. Uncertainty was induced by factors in the environment, especially patient characteristics and medical case complexity, unforeseen incidents and disruptions. Residents’ confidence in their own ability and how well they were supported in aligning their professional identity with the naturally occurring uncertainty of clinical work impacted how well they retained adaptive practices.

## Appendix 1: Tables of analysis

See Tables 3, 4, 5, 6.

*All names are pseudonyms.*

**Table 3:** Epistemic distance as adaptive practice

Actor	Moderator	Consequence	Example
Patients and relatives	Uncertainty	Acquire tests based on gut feeling	In a situation with a female patient who has fallen, the resident (Christina) believes that it is necessary to order an x-ray. She contemplates: “*I don’t quite know if it will show anything, but from previous experience I know, that it is a good guidepost if I have the feeling that the idea will stay in my mind, if I don’t put it at rest before I send her home.”* This shows that her uncertainty is grounded in intuition
		Rigid use of structured history uptake, guideline or algorithm	The resident (Catherine) holds a laminated card in her hand, while questioning the patient and daughter. The patient is not able to respond, and the resident focuses on the daughter, continually consulting her card, in order to follow the guideline. The patient starts to shift in her bed, correcting her and looking around, however, the resident does not notice this growing discomfort of the patient
	Disruptions	Overlook contextual cues	The resident (Catherine) continues to question the daughter to the female patient about her mothers’ medication and chronic obstructive pulmonary disease. The daughter replies and mentions the patients’ memory problems again. The patient interrupts: *“I feel unkempt… I have for some time.”*
			The resident tries to calm her, saying that she doesn’t see her as unkempt at all. The patient looks lost, and it seems to me that the patient is trying to communicate a general discomfort or underlying problem, which the resident doesn’t pick up on
		Explore the problem space	The resident (Daniel) continues to ask questions, but the patient quickly becomes more uncomfortable, moving around in the bed, taking off his duvet, is shaking and visibly uneasy. This prompts the resident to start a physical examination
	Use relatives to gain information	Explore the problem space	The resident (Daniel) concludes this encounter by stating a preliminary treatment plan and asks about the wife’s checklist. The wife hands the resident her phone, where there’s a text message from her daughter. The resident reads the message and asks: *“his head tilts towards the right?”*
Electronic patient journal	Focused read-through	Explore the problem space	The resident (Sophie) sits down to re-read the journal: a geriatric examination has been performed two days prior, which describes that the patient has continuing falls, but that it does not require further examination. She isn't satisfied with that information and goes to find a supervising physician to consult
	Surprising findings in routine read-throughs	Detect knowledge gaps	From the x-rays and the journal entry, the resident (Sarah) concludes that the arm is an uncomplicated fracture, and they move on to the hip. Here the resident is surprised that it looks like a fracture on the pelvis as well, uttering *"oh there was a fracture! I didn’t think so"*
		Explore the problem space	The resident (Christina) checks the journal entry and comments: *“Oh, she was unconscious”*. I ask what she notices, and the resident explains: *“She has chronic pain. There’s a neurological awareness, as she has fallen so we want to check her [blood samples]. There’s risk with falling when the patient is on anticoagulant medication”*
	Results of tests	Seek out professionals with greater knowledge in order to explore the problem space	The resident (Sarah) returns to the office to find a physiotherapist to consult the X-ray pictures. They agree that it looks like a fracture on the pelvis, but that they also need a more thorough physical exam
Supervising Physician	Observing supervising physician's decisions	Explore the problem space	The resident (Maria) observes and asks if the supervising physicians’ actions reflects that the patient is acute, which the supervisor rejects. She continues to ask clarifying questions about the bloodwork and tests, that is being ordered and how she would approach the patient. R8 then suggest hypotheses (not prompted) which the supervisor comment on what could confirm or reject them
	Supporting agency by help identifying possible actions to take	Help identify knowledge gaps and explore the problem space	The resident (R) (Sophie) is sought out by the supervising physician (SP), to confer
			SP: *“how about the falling?”*
			[the resident explains the geriatric examination.]
			SP: *“I would call them to ask if they are sure, since she has multiple fractures in different places.”*
			R: *“but she doesn’t want to have it examined?”*
			SP: *“no, but it indicates a high mortality risk”*
	Praise in supervision	Support agency to explore the problem space	The supervising physician praises the resident (Casper) (nods and gives affirmative utterances), but also leaves the decision up to the resident: *“if you have the least bit of a knot in your stomach, then order an X-ray, so that you are sure it is in place”*. The resident responds that he has confidence in his own treatment decision and goes with his plan of having the nurse redo the sling
	Regulating heuristics	Restrict the opportunity to explore the problem space, because of assumptions of age-related level of functionality	During the residents’ (Maria) conferral with her supervising physician (SP), the SP summarizes her findings, but does not involve the resident in her decision-making process, despite the resident making diagnostic remarks:
			SP: “she is OK on her [vital signs] but she is close to triaging orange… it can be bird or fish… she’s exciting!”
			Resident: “and the age!”
			SP: “yeah, it’s impressive!”
	Disinterest in the resident	Prompt a need for a deeper understanding of the problem space	During the conferring the supervising physician only answers when the resident asks questions. After the conferring, the resident (Maria) seeks out information by saying that there are still things she is unsure of
	Quizzing the resident	Interfering agency in exploring the problem space	The supervising physician continues to look at the screen while going through the blood tests. She verbalizes her reasoning on all values and they talk about possible diagnosis, primarily her proposing them and the resident (Daniel) agreeing by nodding and uttering confirmative sounds. It seems as if she is checking the residents’ knowledge by asking “what can we do with these?” or “what does they tell us?” with the tests that they talk about ordering
Fellow residents or medical students	Using experience to teach	Commit premature closure	The resident (Sarah) explains to the medical student (James) that she had checked the patients’ pelvis and rejects there being a fracture on the hips. She explains that she has encountered this condition on a previous occasion: *“you are not in doubt when there’s a fracture on the hips – the legs are much more concave”* she says and gestures a concave form with her hands

**Table 4:** Self-regulation as adaptive practices

Actor	Moderator	Consequence	Example
Supervising Physician	Authoritative support/taking over	Supervising physician not providing the resident the opportunity to independently monitor levels of knowledge and taking steps to close gaps	The nurse calls to handover the patient to the supervising physician. The resident (Maria) listens in, as she is taking over the patient. The supervising physician comments: *“[the patient] hasn’t been to the hospital in ages”* and continues to order additional blood tests an IV drop and other extra testing. Meanwhile, the resident is noting everything down, but not partake in the decision process of what they need to test for
		Fostering dependent and checking behaviour	During the patient meeting the supervising physician (SP) keeps present but in the background to observe. The resident (Maria), however, continually address the SP in order to check that her strategy correspond to their agreed approach, by asking if her actions are correct. The SP gently corrects and then encourages her to go on
	Answering clarifying questions	Monitoring current levels of knowledge and checking with supervising physician to decrease uncertainty	When asked about how he uses the conferral, the resident (Casper) explains: *"I learn a lot when I get constructive feedback and is corrected. I also always check everything and is very systematic in my approach. If I feel like the conferring with the SP is not good enough, I continue to seek help and asks questions until I feel comfortable with the decision. I don't want to go home with a knot in my stomach."*
	Supporting agency by prompting critical reflection about what symptoms mean	Monitoring level of knowledge, shifting working hypothesis and taking steps to close knowledge gaps	During the conferral, the resident (Daniel) becomes more explorative, asking more open-ended questions, as the supervising physicians helps him realize that it is too early to come up with any tangible hypotheses. The supervising physician argues that it is important to start out monitoring how the patient responds to the initial treatment for dehydration, before moving on to additional testing. The conferring concludes by the resident changing his working hypotheses of UTI and follows the supervising physicians’ hypotheses about dehydration
		Fostering critical reflection and questioning, helping differential diagnostic processing	
		Helping to decide on steps to take in order to address knowledge gaps	
	Modelling	Modelling being aware when they are not adequate to address the problem and how to address the gap in knowledge	The resident (Sophie) is treated an elderly female patient with a multitude of medical problems. It is a challenge for her to get the patient to take part in the clinical examination. After finishing the initial history take, the resident returns to her supervising physician. The supervising physician instructs her to call the department of lung disease, and how to approach it. The supervising physician comments that this patient has turned down examination there before, so the resident should ask the patient first. They look through the journal and the supervising physician mentions that the geriatrician informed her that the patient herself ended the examination process of her tendency to fall. She uses this information to guide the resident on how to motivate the patient
Nurses	Disruptions	Enforcing structure or method	A nurse interrupts the resident (Julie) while taking history on the patient, in order to place a catheter. The nurse struggles with it and brings in several other nurses to help her. While this is going on, the resident explains to me, how it is hard to keep on track when everything becomes chaotic. She worries about forgetting things and how it forces her to become much more rigid in her questioning. After the patient meeting, the resident worries that sticking to a set agenda did not leave much space for follow-up questions, which could have given her some more insight
	Uncertainty	Sharing knowledge, by being aware that they are not yet adequate to address the problem	The resident (Sarah) is treating her second patient of the day, who is challenging. The resident explains to the researcher that when patients are challenging, they often talk with the nurse who is assigned the patient:* "Then you often vent together with the nurse who is on the same patient. You often also consult her after, just to check, if she thought that you chose the right treatment"*
		Consulting in order to close knowledge gaps	The resident (Casper) concludes the physical exam and seeks out the responsible nurse, as he wants to know how the patient's habitual condition is [in regard to the patient's extreme aphasia] and how he was brought to the ED, in order to assess if he is also having some degree of delirium
Patients and relatives	Disruptions	Monitoring levels of knowledge	In the field interview, the resident (Sarah) informs the researcher that she has set procedure that she follows. However, during the history take, the poor state of the patient (P) prompted her to take additional tests, to be sure that the patient wasn’t suffering from blood loss: *“I wanted to know; how affected is he? And what am I sending him home to?”* This was presented in the following exchange between the resident and patient:
			P: *“I don’t feel nauseous”*
			R: *“You don’t feel nauseous?”*
			P: *“No”*
			R: *“Are you dizzy?”*
			P: *“No”*
		Taking steps to close knowledge gaps like ordering tests or discarding irrelevant information	The wife explains that [the patient complains] when he sits upright. The resident (Daniel) makes the patient sit up in the bed in order to checks his back and neck again, but the patient has a hard time understanding what is going on and cannot cooperate. The resident comments that “it is important to at least get that X-ray going”
	Uncertainty	Ordering tests to check for gaps in knowledge	The resident (Sarah) is treating her second patient of the day and orders an X-ray *“to be sure”.* She explains that the patient is sore when putting compression pressure on the hip
	Disruptions: lack of alignment between patient symptoms and working hypothesis	Ordering tests or seeking out help to address gaps in knowledge	Cued by contradicting findings in the physical examination of the patient’s pain, the resident (Christina) re-examines the patients’ swelling. She wants to clarify if he is experiencing any pain in his face, other than locally from where he has broken his jaw and looks under his tongue for any haemorrhages
			The resident comments that *“I do not think you have any new fractures; I think it is the swelling”.* However, she is not satisfied with the adequacy of her own knowledge and calls a specialist in order to confer her findings
Electronic patient journal	Referrals	Cueing monitoring current levels of knowledge	While explaining how he uses referrals, the resident (Daniel) explains to the researcher: *“There’s also specific referrals which calls for specific actions. For example, “OBS pneumonia” or “OBS infection” are often more general indications of an overall worsened habitual condition and then you prepare for anything. Then, you are OBS on everything.”*
	Focused read-through	Helping to take steps to close knowledge gabs or discarding findings/information irrelevant for working hypothesis	The resident (Sophie) re-reads the patient journal and finds that a geriatric examination has been performed two days prior, which describes that the patient has continuing falls, but that it does not require further examination
	Uncertainty	Looking up and comparing prior patient encounters to current patient, in order to monitor levels of knowledge and help decide on steps to take in order to address these	The resident (Sophie) is making decisions on how to get the best picture of the suspected fracture: *“I’m not sure if I should order both shoulder and upper arm, because I don’t know how specific they are… But I had one done on Saturday…”*
			The resident looks up that x-ray and based on that decides that an x-ray of the shoulder would be adequate
Flowcharts or guidelines	Patient complexity	Helping monitoring levels of knowledge and deciding on steps to take in order to address these	The resident (Christina) considers how to examine the arm, where she already has chronic pain and look at the flowchart to decide whether the patient is eligible for CT or not. She orders it
	Unknown symptoms	Helping address knowledge gaps	The resident (Christina) looks up pictures of these nerves in medical handbooks to prepare herself for what to look for, while waiting for the CT results
Resident’s reflection	Uncertainty	Ordering tests to address gaps in knowledge	The resident (Sarah) is treating her second patient of the day, and looks for a more experienced physician, but cannot find one. To be sure before deciding on a treatment, she orders more X-rays
	Patient complexity	Prompting monitoring of levels of knowledge	As the resident (Christina) concludes her conferral with the supervising physician, she sighs and comments: *“so she has to get a lot done”* referring to the complexity of finding out, why the patient has fallen

**Table 5:** Orientation to novel content as adaptive practices

Actor	Moderators	Consequence	Example
Supervising Physician	Not helping the resident structure their conferral	Maintaining a lack of overview and obscuring learning opportunities for the resident	The chaos of the patient encounter carries over to the conferring with a supervising physician, as the reporting becomes sporadic, and the resident (Julie) has difficulty sorting out the relevant information. No shared reflective practice is initiated by the supervising physician. Thus, her need for discussing a patient with the supervising physician becomes more similar to a handover, resulting in the supervising physician giving out orders to the resident
	Formal structures	Employing a safety net to ensure that the resident does not restrict the problem space to fit their competencies	The resident (Christina) explains that she will confer with a supervising physician, whom *“might want to check, himself”*
	Uncertainty	Orienting to novel content and consulting/questioning specialists in order to learn	The resident (R) (Christina) has called an orthopaedic surgeon (OS) specialised in facial bones, to assess the patient. The resident starts by describing the patient and concludes: *“and now he can’t bite down, which he could earlier, despite the fracture.”* She shows the OS the x-ray and description
			OS: *“it is not very large”*
			R: *“no, exactly”*
			OS: *“I can understand that it is hard to see.”*
			The resident asks more questions about the fracture and points to the place, where it is. OS finds it, zoom in and show her by pointing
			R: *“ah yeah there! I can see that.”*
			The resident asks if she can observe his patient examination, and we follow OS to go see the patient. OS takes the lead, and the resident keeps in the background. The resident primarily observes, but OS sometimes describes her decision-making to the resident
Electronic patient journal (EPJ)	Saving patients in task portfolios	Orienting to novel content and identifying learning opportunities by following up on their patient’s progress and outcomes	Back in the office, the resident (Daniel) shows me how he often creates tasks on the patients for himself in EPJ, if he found that the patient was hard to figure out. He explains that this provides learning opportunities, as all patients are handed over to specialized physicians in the afternoon, and therefore, the residents often do not get the opportunity to follow the patient all the way until discharge. This is partly because he is curious, but especially to follow up on the final diagnosis and treatment, in order to learn from specialists’ decisions and see which of his hypotheses and diagnoses were correct and which mistakes he made
	Test results	Identifying learning opportunities and acquiring help from specialists	The resident (Christina) looks at the CT scan and checks with pictures of a typical fracture on the jaws that she looked up earlier. She is puzzled by the picture. She has called for an orthopaedic surgeon to come into the ED to assess the patient, and now comments *“I want to go with [the orthopaedic surgeon] when she comes, just to learn.”*
Online medical handbook	Insufficient available knowledge	Seeking out and identifying learning opportunities in practice	The resident (Christina) orients herself in an online handbook of the local guidelines but can’t find the guideline for a fractured jaw. She moves on to read through an online medical handbook to sort out diagnostics. She notes to herself: *“I have to at least look under his tongue.”*
Physiotherapists	Test results	Treating all information as novel content due to prior restriction of the problem space	The resident (Sarah) returns to the office to find a physiotherapist to consult the X-ray pictures. They agree that it looks like a fracture on the pelvis, but that they also need a more thorough physical examination. The resident goes to performs the physical examination. When consulting the physiotherapist again, she describes that the patient has pain in her lower back as well, warranting another picture of this area to rule out any further fractures

.

**Table 6:** Routine practices in relation to adaptive practices

Actor	Moderators	Consequences	Examples
Supervising Physician	Curating patient encounter	Checking behaviour	The supervising physician and the resident (Maria) agrees to see the patient together, but that it is the resident who takes the lead
			The resident does a final check of the patient in EPJ: *“there’s really not a lot of medications?”*
			The supervising physician redirects the focus: *“no, but she is an obvious geriatric patient”* arguing that this is indicated by the patient’s age
		Transferring biases or heuristics	The supervising physician briefs the resident (Maria) about the patient, emphasizing the fact that the patient does not speak her language, but that the son is present, and adds: *“So that’s another barrier [to consider]”*
	Taking over	Resident reduced to following orders	The resident (Christina) has called an expert to look at her patient, and has followed him in, to observe. When they get back in the office, the expert looks through the x-rays again and informs the resident of his plan, giving her the responsibility of seeing it through, but does not include her, in his decision-making process: *“Have him bite down on a paper to see how his bite is. It has to heal by itself, but he should be aware of the progression. Only eat soft foods and then follow up in two weeks to see if his bite has corrected.”*
			The resident goes to inform the patient
	Ad-hoc conferral	Checking behaviour	The resident (Christina) has finished the patient, but on the way to inform the patient, she runs into her supervising physician. The resident checks the diagnosis again with the supervising physician: *“so there’s no fracture?”*. The supervising physician goes through the description of the x-ray and confirms
	Treatment planning	Checking behaviour	The resident (Anne) has found her supervising physician to confer the patient. The resident starts by presenting the anamneses and then chronologically checks her plan for examination and rationales for her decisions. The supervising physician approves of her decisions
		Enforcing confidence in choices	The resident (Casper) seeks out a supervising physician to confer, despite being certain of his diagnosis. He explains that this is required. He confers his diagnosis and treatment plan and explains that he is quite certain that there is nothing wrong, other than the sling being misplaced, and that a correction would fix the patient’s discomfort. The supervising physician responds by agreeing and praise him for his certainty. The resident responds to this that he now has confidence in his own treatment decision
Nurses	Disruptions	Checking behaviour	The resident (Julie) describes how it is hard to keep on track when everything becomes chaotic, and she worries about forgetting things and how this forces her to become much more methodical in her questioning of the patient
Patients and relatives	Frailty bias in the resident	Stereotypical behaviour (talks louder)	The resident (Sarah) stands close to the patient and talks with an unusually loud and clear voice, despite no indication that the patient has impaired hearing
	Patient symptoms	Abandoning/changing strategies	The patient start complaining about a pain in her head and the resident (Anne) makes a quick physical examination of the head
	Interruptions (e.g., from patient or relatives)	Enforcing structure	The resident (Daniel) continues his systematic approach. The wife comments that she has been given a checklist from their daughter, in order to be able to remember everything. The resident smiles and tries to hold on to his structure: *“let us address that afterwards”*. The wife obliges: *“yes, let us follow your order of business”*
		Anchoring to hypothesis	The resident (Casper) begins a physical examination, being very confident and methodical/systematic in his approach. The patient at then points to his leg and the resident follow his gaze, trying to understand what the patient is trying to communicate. It seems as if the patient is not aware that it is his arm that is broken, as he seems confused that it is restricted. The resident notices this: *“but it is this, that is broken.”* pointing to the arm
		Retrieving information from relatives	The patient doesn’t understand the residents’ (Catherine) question, so the resident repeats herself, but more slowly. She continues to question both the patient and the daughter about the fall while taking notes, but the patient seems tired and unfocused. Shortly after the patient falls asleep again so the resident gives up and focus on retrieving information from the daughter for the rest of the history take
		Neglecting to handover information to relatives/patient	The history take has been continually interrupted by the challenge of communicating with the patient, which leads the resident (Catherine) to forget to inform the daughter of her plan. The resident (R) is about to leave which sparks a concern in the daughter (D):
			D: *“What about the legs?”*
			R: *“yes, we are taking some blood samples”*
			D: *“What about her confusion?”*
			R: *“yes, we are giving her a saltwater IV”*
		Ad-hoc/unstructured information gathering	Because of the daughter’s request for more information about the IV treatment, the resident (Catherine) suddenly takes the hand of the patient*: “ah It might be difficult to put a drop. We’ll see.”*
	Miscommunication	Abandoning strategies	As the patient keeps falling asleep and has a hard time hearing, the resident (Catherine) abandons her history take and moves on to the physical examination, before returning her attention to the daughter, in order to get the full anamneses
	Rigid history uptake/following flowchart	Missing or ignoring contextual cues	The daughter in law mentions that the patient's blood pressure is high, pointing to the monitor. The resident (Christina) does not look, nor comment on the information, and continues to follow her flowchart, in order to examine for the patients’ (P) level of pain. The resident (R) then focuses on the arm:
			R: *“Is this a familiar pain?”*
			P: *“No…”* The resident notes this
			P: *“I’m not usually this pale”*
Electronic patient journal	Information on patients	Rising concern and inducing biases before seeing the patient	The resident (Casper) reads up on any additional data on the patient, and notes that the patient suffers from expressive aphasia, caused by a brain haemorrhage. The resident checks the patient description from the referral to the ED, if the patient is accompanied by a career, but it seems that he isn’t. The resident states a concern about being able to communicate adequately to assess the problem and treat the patient properly
